# Do children receiving care and support have higher rates of school exclusion than their peers?

**DOI:** 10.23889/ijpds.v9i5.2756

**Published:** 2024-09-18

**Authors:** Helen Hodges

**Affiliations:** 1 CASCADE / Cardiff University

The Children, Young People and Education Committee recently held an inquiry to scrutinise the Welsh Government’s progress in delivering its Programme for Government Commitment to ‘explore radical reform of current services for children looked after and care leavers’. A key observation highlighted in their report was that there was a lack of robust data collected about various aspects of the care system. Notably it was highlighted that there is a lack of data collected about permanent exclusions and suspensions from school amongst children looked after in out of home care (CLA). This study was therefore designed to fill this evidence gap.

Using anonymised linked administrative data held in SAIL Databank to identify those receiving care and support (CRCS) from their local authority, rates of permanent exclusion and suspension have been determined for the different groups within the CRCS cohort (CLA, those on the child protection register (CPR) and those receiving support who are neither CLA nor CPR) which have then been compared to their peers without this experience. Factors such as phase of education, setting, free school meal entitlement, having additional learning needs, age and gender have also been considered along with the reason for the suspension or exclusion.

In drawing comparisons with peers, an intersectional approach was adopted to identify disparity.  It is hoped that findings from this student study will stimulate debate and contribute towards the development of changes in Welsh policy.

